# A Relation between Exopolysaccharide from Lactic Acid Bacteria and Properties of Fermentation Induced Soybean Protein Gels

**DOI:** 10.3390/polym14010090

**Published:** 2021-12-27

**Authors:** Xiaoyu Yang, Jiao Feng, Qianqian Zhu, Rui Hong, Liang Li

**Affiliations:** College of Food Science, Northeast Agricultural University, Harbin 150030, China; 15127415162@163.com (X.Y.); feng235246@163.com (J.F.); Qianqianzhu1029@163.com (Q.Z.)

**Keywords:** exopolysaccharides, lactic acid bacteria, soybean protein, gel properties, correlation

## Abstract

Exopolysaccharide (EPS) producing lactic acid bacteria (LAB) is considered to be an effective texture improver. The effect of LAB strains (different EPS production capacity) on physicochemical properties (texture profile, water distribution, rheological properties, and microstructure), protein conformation, and chemical forces of soybean protein gel was investigated. Correlations between EPS yield and gel properties were established. Large masses of EPS were isolated from *L. casei* fermentation gel (*L. casei*-G, 677.01 ± 19.82 mg/kg). Gel with the highest hardness (319.74 ± 9.98 g) and water holding capacity (WHC, 87.74 ± 2.00%) was also formed with *L. casei*. The conversion of β-sheet to α-helix, the increased hydrophobic interaction and ionic bond helped to form an ordered gel network. The yield was positively correlated with hardness, WHC, A_22_, viscoelasticity, and viscosity, but negatively correlated with A_23_ (*p* < 0.05). The macromolecular properties of EPS (especially the yield) and its incompatibility with proteins could be explained as the main reason for improving gel properties. In conclusion, the EPS producing LAB, especially *L. casei* used in our study, is the best ordinary coagulate replacement in soybean-based products.

## 1. Introduction

Soybean protein is a representative plant protein and plays a decisive role in a variety of food systems: (1) increase protein content and maintain amino acid content; (2) provide beneficial physiological components; and (3) provide good processing properties [1]. In the processing properties, more and more concerns are focused on the gelation of soy protein, which not only has a positive effect on the texture of food, but also improves the sensory and flavor by providing the spatial three-dimensional (3D) network structure for preserving food ingredients [1].

Coagulation is considered to be a key step in the formation of soy protein gel. Fermentation by lactic acid bacteria (LAB) stands out from the many solidification methods, except the role of LAB fermentation itself (e.g., extended shelf life, improved sensory properties, and increased nutritional value), EPS produced by LAB plays an indispensable role [2,3]. Li, Li, Chen, Feng, Rui, Jiang, and Dong [4] investigated the in-situ EPS produced by *Lactobacillus plantarum* 70,810 could be used to modify water holding capacity, textural properties, viscosity and flavor of the products. Surber, Spiegel, Dang, Wolfschoon Pombo, Rohm, and Jaros [5] studied the physicochemical and functional properties of cream cheese prepared by three *Lactococcus lactis* strains with different EPS production. The effect of EPS concentration produced by different strains on the texture of the gel is still controversial, as reported by Surber, Mende, Jaros, and Rohm [6], so the influence of EPS characteristics on the hardness of the gel is still valuable.

EPS-producing LAB fermentation induced gel (ELFG) can be considered as a novel hydrogel. According to previous reports, the gels can be classified into water-based hydrogels and oil-based organogels [7]. Organogels are materials composed of structural agents and a non-polar liquid phase (organic compounds) [8]. A series of organogels have been developed and classified based on the nature of organogelators such as lecithin organogels (LOs) [9]. Organogels or LOs could replace solid/hydrogenated fats in the food industry, transfer hydrophobic bioactive substances, nutritional drugs, or model bioactive compounds with medicinal or cosmetic interest [10,11,12].

Hydrogels are also materials that are usually composed of polysaccharides or proteins, but are hydrophilic polymer networks that have the ability to take up large amounts of water molecules because the polymer chain is rich in hydrophilic functional groups [13]. It can be seen that the composition of organogels and hydrogels is different. It is interesting that they can both deliver substances, but the solubilities of the delivery materials also differ.

As a novel hydrogel, ELFGs are prepared by water phase and soybean protein, which are different from organogels and are more effective for the delivery of hydrophilic substances, whether in the food or pharmaceutical industries. The formation process of ELFGs involves acidification, protein hydrolysis, flavor formation, and metabolite production, which is conducive to improving intestinal health, providing active ingredients, improving flavor, and prolonging shelf life [4]. ELFGs should be also applied as food ingredients such as thickeners, stabilizers, and nutritional fortifiers.

The mechanism of improving the properties of fermentation induced soybean protein gels (FSGs) is not clear, especially the effects of EPS properties on gelation properties. Thus, we compared the effects of four LAB strains (with different EPS production ability) on the physicochemical of FSGs as the purpose of this work. The protein conformation and chemical forces were also tested to understand the changes in gel properties. Furthermore, we related the properties of the gel to EPS yield and drew a final heatmap. Based on the results, the most promising LAB strain for the application in soybean protein foods could be identified.

## 2. Materials and Methods

### 2.1. Strains

LAB strains with EPS production ability were sponsored by the College of Food Science, Northeast Agricultural University (Harbin, Heilongjiang, China).

### 2.2. Preparation of Fermentation Induced Gel (FG) and EPS Isolation

A mixed solution was prepared by dissolving 10% SPI and 2% glucose *(w/v)* in deionized water and sterilized (121 °C, 15 min). LAB (4%) was then added into the mixed solution and cultured at 37 °C for a period of time until pH reached 4.5. The fermented soybean protein gel was transferred to a refrigerator (4 °C) and stored for 12 h after fermentation [14]. The fermentation induced gels (FGs) prepared with the “strain” is expressed as “strain-G”. For example, FG made with *L. acidophilus* is shown as “*L. acidophilus*-G”.

The isolation process of EPS is based on our previous study [15]. The EPS yield coefficient (mg/kg) was calculated according to the following formula:

$$\mathrm{Yield}\;{\mathrm{coefficient}}_{\mathrm{EPS}}\left(\mathrm{mg}/\mathrm{kg}\right)=\frac{\mathrm{Dry}\;\mathrm{EPS}\;\mathrm{weight}*\mathrm{EPS}\;\mathrm{content}}{\mathrm{Gel}\;\mathrm{weight}}$$

### 2.3. Texture Profile Analysis (TPA) and Water Distribution

A texture analyzer (Stable Micro Systems Ltd., Surrey, UK) equipped with a P/36R probe was used to determine the parameters and measured with the compression strain of 30%.

The water holding capacity (WHC) of gel was determined by centrifugation (1000× *g*, 15 min, 4 °C) and calculated according to the following formula:

$$\mathrm{WHC}\left(\%\right)=\frac{\mathrm{Weight}\;\mathrm{of}\;\mathrm{gel}\;\mathrm{after}\;\mathrm{centrifugation}}{\mathrm{Weight}\;\mathrm{of}\;\mathrm{intial}\;\mathrm{gel}}\times 100\%$$

Transverse relaxation time (T_2_) measurements were carried out with a NMR spectrometer (Niumag Co. Ltd., Shanghai, China) at 25 °C [16].

### 2.4. Rheological Analysis

The rheological properties were measured with an AR2000ex rheometer (TA Instruments Ltd., New Castle, DE, USA) equipped with 40 mm diameter stainless steel parallel plates. The effect of EPS producing LAB strains on the rheological properties (including) were investigated. Frequency sweep was carried out and apparent viscosity (η) was also recorded. Storage modulus (G′) during cooling (lowered from 37 to 4 °C) was also monitored. G′ and G″ dependence of f was fitted by power law model equations as follows:

$$G=K\times f^{n}$$

where *K* stands power law constants (Pa s^n^), and n stands exponents.

### 2.5. Scanning Electron Microscopy (SEM)

A S3400 SEM (Hitachi, Tokyo, Japan) was used to observe the microstructure of fermented soybean gels that were cut, fixed, dehydrated, and sprayed before observation.

### 2.6. Raman Spectroscopy

All spectra were recorded from 400 to 2800 cm^−1^ using a DXR2 Raman spectrometer (Thermo Nicolet Inc, Waltham, MA, USA) at 25 °C. Spectral resolution was 1 cm^−1^, laser power was 100 mW, and exposure time was 30 s.

### 2.7. Chemical Forces of Gel

The gel samples were mixed with different denaturing solvents to acquire corresponding chemical forces. The results were expressed by the protein content in denatured solvent.

### 2.8. Statistical Analysis

The data were measured at least three times. The results were evaluated by analysis of variance (ANOVA) using SPSS software version 22 (IBM software, Armonk, NY, USA). Post hoc tests were conducted by Tukey’s test (*p* < 0.05). A Pearson correlation test was performed to explore the relationship between the yield of EPS and the gel properties.

## 3. Results and Discussion

### 3.1. Acidification and EPS Yield

**Figure 1:**
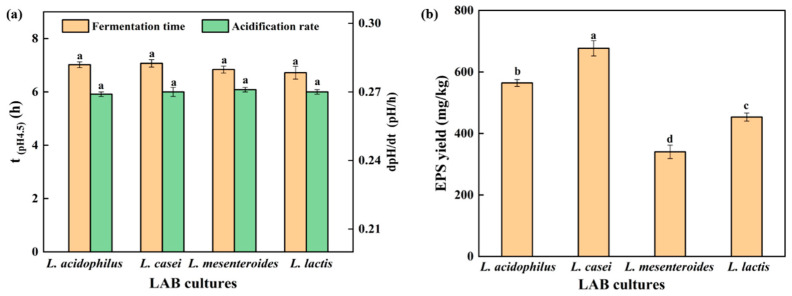
(**a**) Time when the pH value reached 4.5 and acidification rate (until pH = 4.5); (**b**) EPS yield. The values ^(a–d)^ show significant differences (*p* < 0.05).

Acidification rate is an important factor affecting the texture of gel, and increased acidification rate could reduce gel hardness by reducing the arrangement and aggregation time of soybean protein [17]. The acidification rate (dpH/dt) of the SPI gel is shown in Figure 1a. No significant difference in acidification rate between strains was found (*p* < 0.05). In addition, the fermentation time (t_(pH4.5)_), defined as the time for the soy protein to reach the pH of 4.5, was approximately 7 h for all strains (Figure 1a). Large masses of EPS were isolated from *L. casei*-G (677.01 ± 19.82 mg/kg) compared with those from *L. acidophilus* (564.21 ± 15.51 mg/kg), *L. mesenteroides* (340.38 ± 20.67 mg/kg), and *L. lactis* (453.36 ± 30.60 mg/kg), respectively (Figure 1b). The structure of EPS has been characterized in our previous studies [15,18].

### 3.2. Texture Characteristics

Texture characteristics have an important impact on the acceptability of the gel and are shown in Table 1. No obvious differences among gels in cohesiveness were found (*p* < 0.05). Pang, Xu, Zhu, Li, Bansal, and Liu [19] also found that cohesiveness was not affected by the EPS yield. The springiness, gumminess, and chewiness of MEPS were the lowest. The lowest springiness in *L. mesenteroides*-G may be attributed to the lowest WHC, which is shown in Table 1. The relationship between springiness and WHC was supported by the results of Ayyash, Abu-Jdayil, Hamed, and Shaker [20]. EPS yield could improve the hardness of the soy protein gels, which may be attributed to the compact network caused by EPS and soy protein. This could explain why *L. casei*-G had significantly higher hardness than those of the other gels.

### 3.3. WHC and LF-NMR

WHC stands for the capability of the gel to held all or part of its own moisture. The WHC of *L. casei*-G (87.74 ± 2.00%) was the highest (*p* < 0.05) compared with *L. acidophilus*-G (79.26 ± 3.75%), *L. mesenteroides*-G (69.29 ± 1.88%), and *L. lactis*-G (58.99 ± 0.81%), which was linked with the EPS production ability. EPS could positively influence the texture and WHC via the “filler effect” [19].

**Figure 2:**
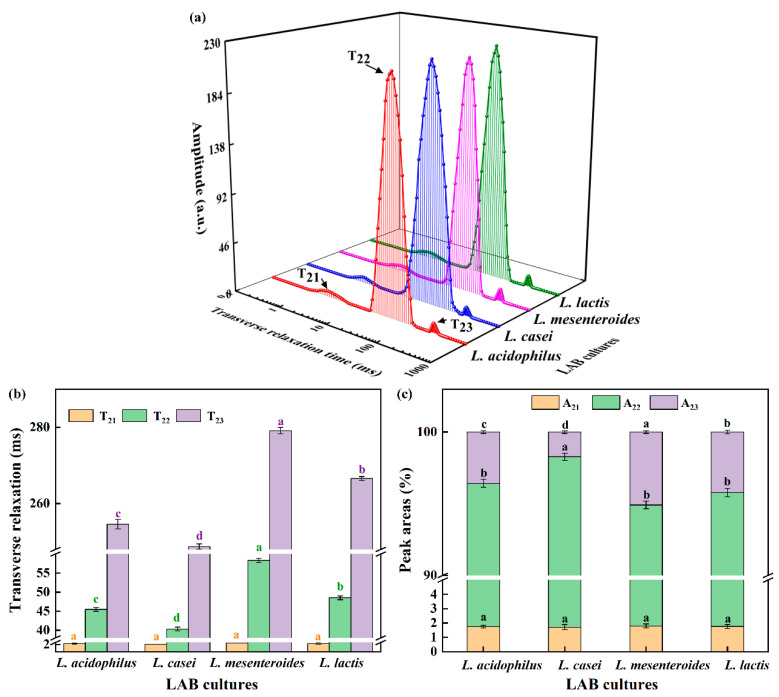
(**a**) Transverse relaxation time curves, (**b**) transverse relaxation times, and (**c**) peak areas of fermentation induced gels. The values ^(a–d)^ show significant differences (*p* < 0.05).

The transversal relaxation time curve is often used to assess differences in water exchange and could reflect denaturation and aggregation [21]. In Figure 2a, T_2_ distribution curves had three peaks, which were also observed in emulsion gels stabilized by SPI and pectin [22]. T_21_ in 1–4 ms stands for binding moisture, T_22_ in 10–200 ms represents fixed water, and T_23_ in 200–400 ms means unbound water. Furthermore, the A refers to the area of the individual peak in T_2_ distribution curves, as shown in Figure 2c. According to Figure 2b,c, there was no significant difference in T_21_ and A_21_ between samples, which showed that the binding moisture’s properties was not dependent on the strains. The trends of T_22_ were similar to that of T_23_. Yang, Zhou, Guo, Feng, Wang, Wang, Ma, and Sun [23] reported that shorter relaxation times were conducive to the combination between water and proteins, so the lowest T_22_ in *L. casei*-G reflected that more free water was retained in the gel structure caused by the binding of water to SPI promoted by EPS. It is worth noting that the A_22_ value was highest, but the A_23_ value was the lowest when the fermentation strain was *L. casei*. The conversion of unbound water to fixed water in the soybean protein gel network resulted in increased WHC of *L. casei*-G (Table 1).

### 3.4. Rheological Properties

**Figure 3:**
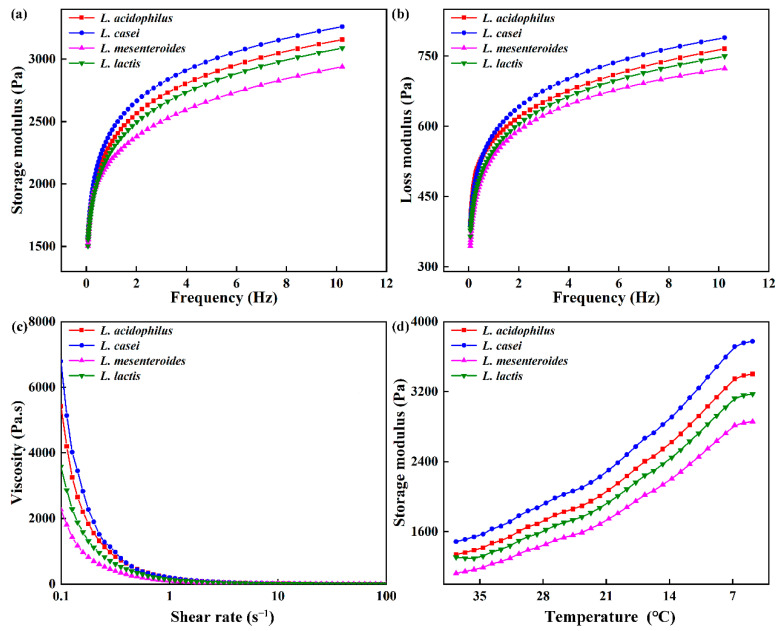
Rheological properties of fermentation induced gels. (**a**) Storage modulus (G′) and (**b**) loss modulus (G″) with frequency, (**c**) viscosity, and (**d**) storage modulus during cooling.

Rheological properties of ELFG were studied. Frequency sweep is usually used as an indicator of gel deformation over time. G′ and loss modulus (G″) of FGs rose with increasing frequency (f), and G′ was higher than G″ (tan δ < 1) during the tested frequency range (Figure 3a,b). All gels exhibited viscoelastic characteristics and strong frequency dependency. *L. casei*-G had the highest G′ at 1 Hz (2434.22 ± 14.33 Pa, *p* < 0.05) (Table 2), which indicated the largest crosslinking degree in the *L. casei*-G. However, no significant change in the tan δ was observed, indicating similar viscoelastic network properties within the LVR of all FGs. The slope of log (G′) vs. log (f) and slope of log (G″) vs. log (f) were found to be higher in the *L. casei*-G than that in *L. mesenteroides*-G, indicating that modulus for the former was more sensitive to frequency. Furthermore, values of slope were in line with values reported for fermentation induced pea protein gel [24]. Good fits were found for the variation trends of G′ and G″ with f (R^2^ > 0.974). Furthermore, the *L. casei*-G had significantly higher K′ (2487.70 ± 15.43 Pa) than those of the others, which was in agreement with the K″ (129.34 ± 1.19 Pa). Figure 2c shows that FGs showed the shear thinning phenomenon. Li, Li, Chen, Feng, Rui, Jiang, and Dong [4] also found this result in fermentation induced soymilk gels, where the decrease in gel viscosity was due to decreased water holding capacity. The *L. casei*-G exhibited higher apparent viscosity than those of the other gels (η_0_ = 6793.57 ± 35.49 mPa s; η_50_ = 2.00 ± 0.08 mPa s), which was in agreement with the WHC results (Table. 1).

The G′ values of the gel increased during the cooling process (Figure 3d), where a similar result was reported [19]. The rising G’ is on account of the drop in the number or strength of the hydrophobic bonds inside each protein particle during the cooling period. The highest G′ was found in *L. casei*-G, which could be due to the EPS produced by *L. casei* forming a denser network via van der Waals forces and electrostatic repulsion between EPS and protein [25]. This agreed with the results obtained during the frequency range (Figure 2a,b).

### 3.5. Microstructure

**Figure 4:**
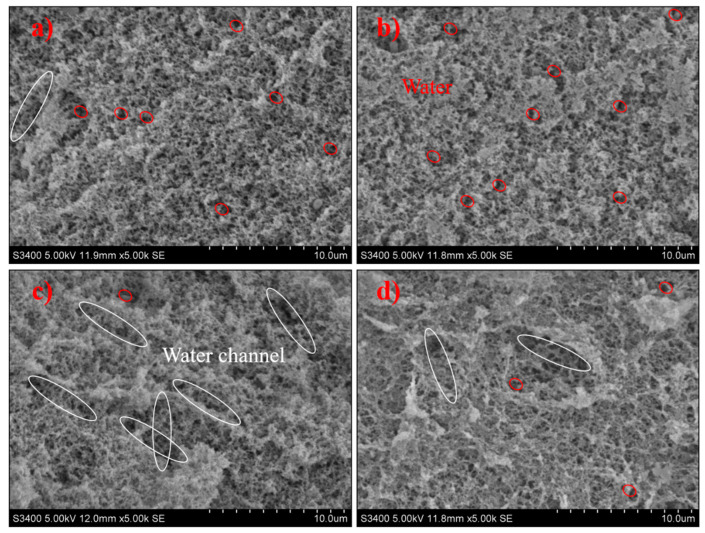
SEM micrographs of fermentation induced gels acidified with (**a**) *L. acidophilus*, (**b**) *L. casei*, (**c**) *L. mesenteroides*, or (**d**) *L. lactis*. White ellipse refers to water channel, red ellipse refers to water molecule.

*L. casei*-G has a dense, uniform, and smooth 3D network structure with smaller holes (Figure 4). However, the microstructure of *L. mesenteroides*-G was crisscrossed by water channels, which would impede the aggregation of protein and lead to the breakdown in the gel network, and larger irregular pores could be found in the coarse and loose 3D network. Water distribution was determined by microstructures; the fluidity of water was restricted by small pores in the 3D network, and vice versa. Larger pores in the coarse gel network structure of *L. mesenteroides*-G increased the water loss depicted in Table 1, which was in accordance with the findings in [26]. The compact structure may be caused by the larger cross-linking of EPS and protein [27]. However, this was inconsistent with the results of [28], who reported that EPS-producing cultures might induce an open structure. According to Pang, Xu, Zhu, Li, Bansal, and Liu [19], the phase separation stage and “filler effect” stage was found. Higher gel hardness (Table 1), smaller water loss (Table 1 and Figure 2), and compact structure (Figure 4) were the main features in the later stage.

### 3.6. Raman Spectral Analysis

**Figure 5:**
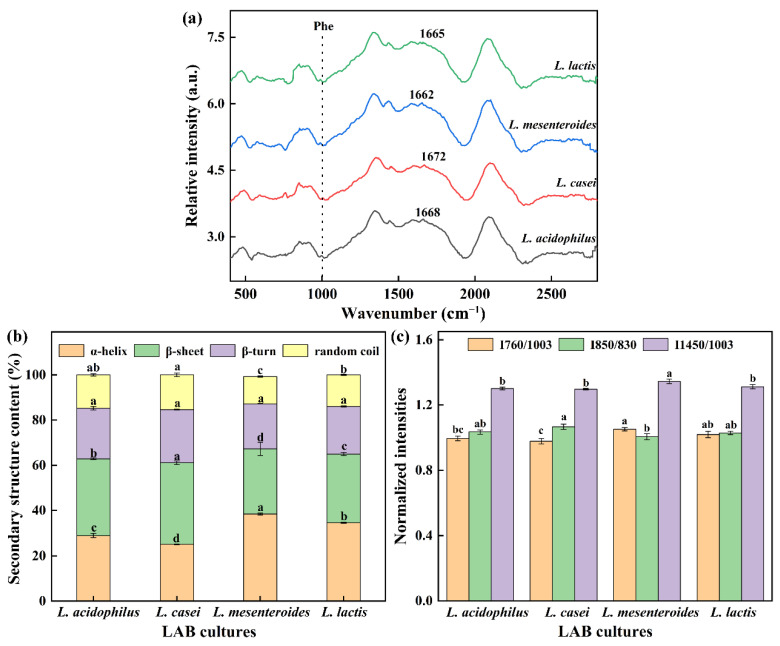
(**a**) Raman spectrum between 400 and 2800 cm^−1^ of gels, (**b**) protein secondary structure, (**c**) normalized intensities of the 760, 850, and 1450 cm^−1^ band. The values ^(a–d)^ show significant differences (*p* < 0.05).

Raman spectroscopy is a powerful tool to explore secondary structure and local environments of soybean protein. The amide I characteristic peak of the gels (except *L. mesenteroides*-G) was at 1672–1665 cm^−1^. It was found that β-sheet was the main secondary structure in FGs due to the characteristic peak located in the β-sheet range (1680–1665 cm^−1^). In addition, the amide I regions are often used to assess secondary structure changes as they contain characteristic peaks of 1658–1650 cm^−1^ (α-helix), 1680–1665 cm^−1^ (β-sheet), and 1665–1660 cm^−1^ (random coil). Figure 5b shows the quantitative analysis results of the amide I band. No significant variation for β-turn proportion in all groups was found, and the similar indifference for random coil was also observed in *L. casei*-G and *L. acidophilus*-G. However, significant changes in the α-helix and β-sheet among groups were found. Our studies found that the enhancement in texture properties and WHC were correlated with decreased α-helix content but increased β-sheet proportion, which had been also observed by Zhuang, Wang, Jiang, Chen, and Zhou [29].

Exposure of tryptophan (Trp) residues in hidden or lyophobic microenvironments to polar aqueous solvents may lead to the reduction of 760 cm^−1^ intensity [29]. Compared with the *L. mesenteroides*-G and *L. lactis*-G, the I760/1003 intensity of *L. casei*-G significantly decreased (*p* < 0.05). The doublet bands located near 830 and 850 cm^−1^ are used to monitor the local environment around tyrosine residues and reflect the changes in hydrogen bonds [30]. The intensity of I850/830 ranged from 1.00 to 1.07. In Figure 5c, the strength of I1453/1003 of *L. mesenteroides*-G had a maximum value (*p* < 0.05), which might be due to the decreased hydrophobic interaction caused by the aliphatic residues embedded in the hydrophobic environment.

### 3.7. Chemical Force Analysis

The changes in protein structure inevitably affect the interaction between protein molecules and the interactions have important effects on gelation properties. Usually, NaCl is used to destroy electrostatic interaction, urea can destroy hydrogen bonds and hydrophobic interactions, and β-ME is a disulfide bond breaker [31].

Molecular forces in gels induced by fermentation with different strains are shown in Table 3. The solubility in (S4-S3) was significantly higher than that in the other three adjacent solvents (*p* < 0.05), indicating that the major force to hold the gel structure was hydrophobic interaction. The chemical forces of ELFGs are hydrophobic interaction > ionic bond > disulfide bond > hydrogen bond. Wang, Shen, Jiang, Song, Liu, and Xie [32] also reported that hydrophobic interactions and ionic bonds play major roles in maintaining the protein-polysaccharide gel systems. When the pH value of protein is close to the isoelectric point of protein, it is easy to denature and aggregate, thus its low solubility leads to the reduction in intermolecular hydrogen bond formation, which explained the lowest hydrogen bond in the gel system. As depicted in Table 3, the increased protein solubility caused the improvement in gel properties. As reported in a previous study, a fine and close 3D structure was formed in the presence of higher protein solubility, and the structure provides the appropriate space for water [31].

### 3.8. Correlation Analysis

FGs made with strains selected in this study had similar fermentation time and acidification rate (Figure 1, *p* < 0.05); the difference in gels could mostly be due to the production of EPS. Hence, the correlations between EPS yield and gel textural, LF-NMR spectroscopic, and rheological characteristics under the LAB fermentation were evaluated, and the correlation diagram was established. This map is represented by the Pearson correlation coefficient (R^2^) between −1 and 1, and the color code was used to better understand the degree of correlation. The yield was assumed to be responsible for different gelation properties, and was used to establish correlations.

**Figure 6:**
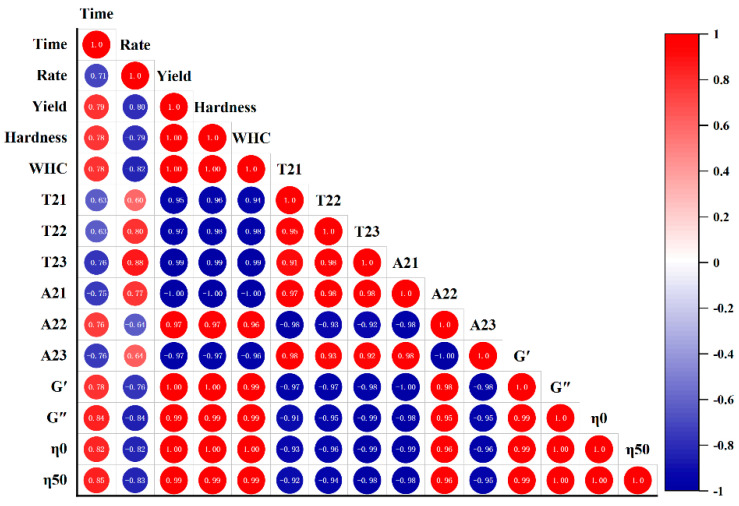
Correlation analysis between the EPS yield and gel hardness, WHC, LF-NMR spectroscopic, and rheological characteristics of the fermentation induced gels.

In Figure 6, no correlations between fermentation time or acidification rate and the gels’ parameters were found. Yield was positively correlated with hardness (0.998), WHC (0.999), A_22_ (0.972), G′(0.995), G″(0.993), η_0_(0.996), and η_50_ (0.994), but negatively correlated with A_23_ (−0.971) (*p* < 0.01). Fixed moisture increased with the increase in EPS hydrophilic groups, which could link with water [33]. EPS enhanced the network structure of FGs, thereby increasing WHC [34] and gel hardness [35]. EPS interacting with proteins could be used as active fillers and increase the viscoelastic modulus [33].

More and more studies have demonstrated the effect of EPS’s macromolecular properties on gel properties. The high M_w_ EPS could strengthen WHC and hardness by interacting with soybean protein [25]. Hassan [36] also found that if the EPS held higher M_w_, hardness was strengthened. Additionally, EPS with high M_w_ may influence protein aggregation and network formation [37]. A positive correlation between the viscosity (η_0_ and η_50_) and high M_w_ EPS production was also reported [4]. G′ increased as the M_w_ increased [38]. Charged EPS increased apparent viscosity (η_0_ and η_50_) by increasing intramolecular repulsions within the polymer chains and electrostatic interactions between anionic EPS and soybean proteins [39]. It was also found that the net negative charge led to stronger hydrophobic interaction, which contributed to an increase in hardness [40]. The above studies provide a direction for our future research.

Interestingly, correlations were also detected between gel hardness, viscoelasticity, and hydrodynamic properties. Gel hardness was positively related to the WHC (0.998) and Xi, Liu, McClements, and Zou [41] also believed that T_22_ had a strong negative correlation with gel viscosity (η_0_), and the correlation coefficient was −0.958; A_22_ and G′ showed a strong positive correlation (0.985); T_2_ decreased with the increase in hardness. This was also found in [42], who also said that the correlations above-mentioned between the LF-NMR spectroscopic characteristics and the rheological properties. T_2_ was negatively correlated with G′, which was consistent with the results of [43].

## 4. Conclusions

Our findings confirmed the key significance of EPS production in the physicochemical properties, protein conformation, and chemical forces of FGs. The outcomes showed that hardness, viscoelastic, and apparent viscosity of gels induced with *L. casei* was the highest. The Raman spectra and chemical forces showed that conversion of the β-sheet to α-helix and the increased hydrophobic interaction, resulted in an order, smooth, and uniform 3D network structure. Yield could be regarded as the main reason, which was responsible for the enhancement of the gels’ characteristics. The influence of EPS on the gel properties depends not only on the EPS own macromolecular properties, but also on the ability to interact with proteins. Overall, the results indicate that EPS producing LAB could be used wisely to modify the gel properties of soybean proteins and to form novel protein gels.

## Figures and Tables

**Table 1:** Texture profile analysis parameters and WHC of gels.

	*L. acidophilus*	*L. casei*	*L. mesenteroides*	*L. lactis*
Hardness (g)	278.60 ± 8.93 ^b^	319.74 ± 9.98 ^a^	200.99 ± 6.41 ^d^	242.29 ± 8.74 ^c^
Springiness	0.76 ± 0.03 ^ab^	0.80 ± 0.01 ^a^	0.65 ± 0.02 ^c^	0.74 ± 0.01 ^b^
Cohesiveness	0.46 ± 0.02 ^a^	0.48 ± 0.02 ^a^	0.47 ± 0.03 ^a^	0.48 ± 0.02 ^a^
Gumminess	114.15 ± 12.34 ^b^	108.45 ± 2.20 ^b^	66.41 ± 5.59 ^c^	140.87 ± 7.90 ^a^
Chewiness	89.58 ± 12.52 ^b^	77.07 ± 3.88 ^b^	46.85 ± 13.62 ^c^	116.35 ± 6.31 ^a^
WHC (%)	79.26 ± 3.75 ^b^	87.74 ± 2.00 ^a^	58.99 ± 0.81 ^d^	69.29 ± 1.88 ^c^

Values with a different superscript letter ^(a–d)^ were significantly different (*p* < 0.05).

**Table 2:** Rheological parameters from flow curve and frequency sweep of gels.

	*L. acidophilus*	*L. casei*	*L. mesenteroides*	*L. lactis*
G′, at 1 Hz	2344.97 ± 18.06 ^b^	2434.22 ± 14.33 ^a^	2205.23 ± 17.74 ^d^	2278.50 ± 25.43 ^c^
G″, at 1 Hz	574.28 ± 15.05 ^ab^	586.24 ± 11.64 ^a^	540.40 ± 10.41 ^c^	552.92 ± 13.12 ^bc^
Tan δ, at 1 Hz	0.250 ± 0.015 ^a^	0.240 ± 0.013 ^a^	0.265 ± 0.013 ^a^	0.256 ± 0.005 ^a^
Slope of log (G′) vs. log (f)	0.130 ± 0.004 ^a^	0.160 ± 0.008 ^a^	0.124 ± 0.005 ^b^	0.139 ± 0.005 ^a^
Slope of log (G″) vs. log (f)	0.139 ± 0.009 ^ab^	0.146 ± 0.007 ^a^	0.110 ± 0.005 ^b^	0.139 ± 0.003 ^ab^
K′	2316.02 ± 8.74 ^b^	2487.70 ± 15.43 ^a^	2197.95 ± 10.08 ^d^	2257.28 ± 19.49 ^c^
n′	0.138 ± 0.012 ^a^	0.142 ± 0.011 ^a^	0.123 ± 0.009 ^a^	0.138 ± 0.002 ^a^
R′^2^	0.999	0.996	0.996	0.999
K″	118.32 ± 1.31 ^b^	129.34 ± 1.19 ^a^	90.81 ± 0.95 ^d^	99.73 ± 0.62 ^c^
n″	0.224 ± 0.023 ^ab^	0.25 ± 0.009 ^a^	0.198 ± 0.007 ^b^	0.216 ± 0.015 ^ab^
R″^2^	0.995	0.997	0.974	0.997
η_0_ (mPa s)	5419.14 ± 73.19 ^b^	6793.57 ± 35.49 ^a^	2265.62 ± 57.63 ^d^	3577.73 ± 67.86 ^c^
η_50_ (mPa s)	1.66 ± 0.10 ^b^	2.00 ± 0.08 ^a^	0.80 ± 0.01 ^d^	1.10 ± 0.00 ^c^

Values with a different superscript letter ^(a–d)^ are significantly different (*p* < 0.05).

**Table 3:** Molecular force changes involved in gels.

(mg/mL)	*L. acidophilus*	*L. casei*	*L. mesenteroides*	*L. lactis*
S2–S1 Ionic bond	8.48 ± 0.14 ^Bb^	9.50 ± 0.18 ^Ba^	7.08 ± 0.11 ^Bd^	7.69 ± 0.17 ^Bc^
S3–S2 Hydrogen bonds	3.12 ± 0.03 ^Cb^	3.23 ± 0.04 ^Da^	2.93 ± 0.03 ^Cc^	3.08 ± 0.05 ^Cb^
S4–S3 Hydrophobic interactions	24.57 ± 1.94 ^Ab^	29.83 ± 1.74 ^Aa^	20.58 ± 1.25 ^Abc^	22.10 ± 1.30 ^Ac^
S5–S4 Disulfide bonds	6.56 ± 0.10 ^Bb^	6.97 ± 0.10 ^Ca^	6.01 ± 0.12 ^Bc^	6.23 ± 0.11 ^Bc^

^A–D^ Indicate significant differences between the same column. ^a–d^ Represent the differences between the same line.

## Data Availability

The data presented in this study are available on request from the corresponding author.
